# MicroRNA as Potential Biomarkers of Platelet Function on Antiplatelet Therapy: A Review

**DOI:** 10.3389/fphys.2021.652579

**Published:** 2021-04-15

**Authors:** Pamela Czajka, Alex Fitas, Daniel Jakubik, Ceren Eyileten, Aleksandra Gasecka, Zofia Wicik, Jolanta M. Siller-Matula, Krzysztof J. Filipiak, Marek Postula

**Affiliations:** ^1^Department of Experimental and Clinical Pharmacology, Medical University of Warsaw, Center for Preclinical Research and Technology, Warsaw, Poland; ^2^First Chair and Department of Cardiology, Medical University of Warsaw, Warsaw, Poland; ^3^Centro de Matemática, Computação e Cognição, Universidade Federal do ABC, São Paulo, Brazil; ^4^Department of Cardiology, Medical University of Vienna, Vienna, Austria

**Keywords:** miRNA, platelets, platelet reactivity, antiplatelet therapy, biomarker

## Abstract

MicroRNAs (miRNAs) are small, non-coding RNAs, able to regulate cellular functions by specific gene modifications. Platelets are the major source for circulating miRNAs, with significant regulatory potential on cardiovascular pathophysiology. MiRNAs have been shown to modify the expression of platelet proteins influencing platelet reactivity. Circulating miRNAs can be determined from plasma, serum, or whole blood, and they can be used as diagnostic and prognostic biomarkers of platelet reactivity during antiplatelet therapy as well as novel therapeutic targets in cardiovascular diseases (CVDs). Herein, we review diagnostic and prognostic value of miRNAs levels related to platelet reactivity based on human studies, presenting its interindividual variability as well as the substantial role of genetics. Furthermore, we discuss antiplatelet treatment in the context of miRNAs alterations related to pathways associated with drug response.

## Introduction

Platelets are anuclear morphotic element of the blood, derived from megakaryocytes with a circulating lifespan of 7–10 days (Davì and Patrono, 2007). They are responsible for the primary hemostasis binding to the subendothelial extracellular matrix exposed after vessel damage. Contact with endothelial adhesion molecules leads to platelet activation, secretion of mediators contained in their granules, and aggregation (Pordzik et al., 2018). Platelet activation is also promoted by the interaction between platelet surface membrane receptors and agonists, such as thromboxane A2 (TXA2), adenosine diphosphate (ADP), and thrombin (Davì and Patrono, 2007). Moreover, platelets cooperating with endothelial cells and leukocytes are also players in the inflammatory response (Paniccia et al., 2015). Although anucleated platelets do not have genomic DNA in nature, *de novo* gene transcription in their cytoplasm relies on numerous microRNAs (miRNAs, miRs), messenger RNAs (mRNAs), long non-coding RNAs, and proteins that modulate their expression (Provost, 2017).

MiRNAs are small (18–25 nucleotides), non-coding RNAs that influence various aspects of cellular functions through regulation of gene expression (Eyileten et al., 2020; Gasecka et al., 2020; Soplinska et al., 2020). The essence of their regulatory effects is the ability to recognize and specifically bind into the 3’ untranslated region (3’UTR) of mRNAs (Jarosz-Popek et al., 2020). One miRNA can bind and regulate more than one target gene, and one single target can simultaneously be regulated by several miRNAs (Wolska et al., 2020; Zareba et al., 2020). Moreover, one single miRNA may target different mRNAs that encode proteins from the same signaling pathway, thus enriching its own regulatory capacity (Kaudewitz et al., 2016; Basak et al., 2019; Jakubik et al., 2021). It was reported that miRNAs are abundantly expressed in platelets, which contain protein enzymes, such as Dicer and argonaute 2 (Ago2) participating in the conversion of megakaryocyte-originating pre-miRNA into the mature miRNA (Provost et al., 2002; Elgheznawy et al., 2015; Pordzik et al., 2019). MiRNAs play an important role in platelet function and platelet reactivity pathways. Some of them can target mRNAs coding proteins participating in the activation and aggregation, and therefore regulate their expression. Even though the majority of miRNAs are found intracellularly, a substantial amount appears in the extracellular space, including blood and other body fluids (Weber et al., 2010). Circulating miRNAs can be detected, i.e., in plasma, serum, or whole blood, and they can be used as diagnostic and prognostic novel biomarkers as well as novel therapeutic targets (Eyileten et al., 2018; Sabatino et al., 2019). There are three pathways through which miRNAs can get to the extracellular space—passive leakage from damaged cells, active secretion in the microvesicles, such as platelet-derived extracellular vesicles (PEVs), or through the active secretion in an RNA-binding protein-dependent pathway (Zhao et al., 2019).

Herein, we provide a brief description of methods of platelet function assessment and a comprehensive presentation of the studies that investigated various miRNAs related to platelet functions and platelet reactivity on antiplatelet therapy. Moreover, we present an up-to-date review on diagnostic and prognostic value as well as therapeutic potential of miRNA levels related to platelet reactivity based on human studies. In this review, we focus on the influence of antiplatelet drugs on the platelet-miRNAs expression, though we hypothesize that identification of specific patterns in miRNAs expression, changes in their levels could help to forecast the risk of occurrence of high on-treatment platelet reactivity (HTPR) and major adverse cardiovascular events (MACE).

## Determination of Platelet Function on Antiplatelet Therapy

Platelets are crucial for both hemostasis and thrombosis; thus, platelet function tests are an important clinical tool for the identification of patients with bleeding disorders, monitoring the response to antiplatelet treatment, in the evaluation of perioperative hemostasis, and also in transfusion medicine (Paniccia et al., 2015). There are various approaches for platelet function assessment, and most of them focus on the measurements carried out after platelet activation caused by agonist administration (Koltai et al., 2017; Ostrowska et al., 2019). Despite the wide range of available tests, some are discussed below, new, more sensitive and specific biomarkers are needed, especially for the monitoring of antiplatelet therapy (Gross et al., 2016). It has been revealed in some research that sP-selectin or CD40L might serve as such biomarkers, but their detectability time in general circulation is short (Yun et al., 2016). Cautious monitoring of platelet function while antiplatelet therapy is administered could help to prevent the adverse outcomes (Aradi et al., 2010; Lenk and Spannagl, 2013). In this aspect, miRNA may be considered an interesting and potentially promising alternative for currently used methods of platelet function assessment.

Nowadays, in terms of diagnostic tools for the inherited platelet function disorders, International Society on Thrombosis and Hemostasis(ISTH) recommends primarily light transmission platelet aggregometry (LTA) and flow cytometry as the most important and valuable methods. Other tests for platelet function assessment, such as VerifyNow, vasodilator-stimulated phosphoprotein-phosphorylation (VASP-P), and multiplate electrode aggregometry (MEA) are also used for the monitoring of antiplatelet treatment; however, it is LTA that is a gold standard for platelet function assessment (Paniccia et al., 2015). This time-consuming and technically-challenging test is based on the transmission of light through platelets in a suspension of platelet-rich plasma (PRP), washed or gel-filtered platelets. Platelets aggregate in the presence of an agonist, which leads to the increase in light transmission. ADP, epinephrine, collagen, proteinase-activated receptor 1 activation peptide (PAR1-AP), TXA2, arachidonic acid (AA), or the agglutinating agent ristocetin can be used as an agonist. Clinically, LTA is a recommended tool for the diagnosis of subjects with bleeding disorders—it is listed next to flow cytometry, as a first-step test in a diagnosis of inherited platelet dysfunctions. Moreover, according to the ISTH Scientific and Standardization Committee guidelines, LTA can be used for the identification of thrombosis risk subjects as well as monitoring of those on antiplatelet therapy but only for the scientific purposes (Cattaneo et al., 2013; Gresele and Subcommittee on Platelet Physiology of the International Society on Thrombosis and Hemostasis, 2015; Paniccia et al., 2015). In most clinical studies, tests other than LTA were performed to assess platelet functions. One of the most commonly used tests for monitoring patients on antiplatelet therapy is the VerifyNow system. In this test, platelets are activated with agonists and optical detection is used to check the results. It can be performed with the use of whole blood samples. Two assays sensitive for specific drugs—acetylsalicylic acid (ASA) and P2Y_12_ receptor inhibitors—are available. Rate and degree of platelet aggregation are presented as aspirin reaction units or P2Y_12_ reaction units, respectively. The VerifyNow system was proved to be useful in classifying patients as at a high risk of MACE, not responsive to ASA or P2Y_12_ inhibitors (Gachet and Aleil, 2008; Postula et al., 2013; Paniccia et al., 2015). A flow cytometry test, VASP-P assay, can be used, next to VerifyNow, to verify the effect of the platelet P2Y_12_ antagonists in whole blood samples (Gachet and Aleil, 2008). This is the most specific test available for the assessment of P2Y_12_ receptor blockade and shows some significant advantages—one of those is the fact that ASA and other medications, like glycoprotein IIb/IIIa (GPIIb/IIIa) antagonists do not impact the results. However, in contrast to LTA, the results of VASP-P assay will reflect only one platelet activation pathway (Bagoly et al., 2013; Mingant et al., 2018). Another test—MEA is a fast diagnostic method that enables the assessment of platelet function in the whole blood samples. In order to perform MEA, an agonist of a particular pathway of activation is added to whole blood samples with anticoagulant—citrate or hirudin. What is important, MEA can be used in the assessment of platelet functions of patients on various antiplatelet drugs—ASA, P2Y_12_ inhibitors, or GPIIb/IIIa receptor blockers (Pluta et al., 2018).

As it was demonstrated, different tests are available for the measurement of platelet reactivity based on different activation pathways (Paniccia et al., 2015). Thus, the results of individual tests are not directly comparable (Siller-Matula et al., 2015). It should be emphasized that there is still not enough data supporting the usefulness of any laboratory tests in the monitoring of antiplatelet treatment (Cattaneo et al., 2013). According to the European Society of Cardiology (ESC) 2020 guidelines, platelet function testing has a low level of recommendation (class IIb) in the risk assessment of de-escalation of antiplatelet treatment (Collet et al., 2020). The recommended tests in the monitoring of antiplatelet treatment with P2Y_12_ inhibitors are VerifyNow P2Y_12_ assay, the Multiplate device with the ADP kit, and the VASP assay. Only in the absence of standardized assays, LTA is recommended (Lenk and Spannagl, 2013). The available platelet function tests are insufficient for reliable detection of HTPR patients (Roule et al., 2018); therefore, novel methods and markers for the platelet function assessment could be useful in clinical practice.

### Methods of Determination of Platelet-Related miRNAs in the Context of Antiplatelet Treatment

Due to numerous limitations of currently used platelet function test, miRNAs could be a promising candidate, as they are stable in biological samples, although their low levels constitute a challenge for their quantification. For miRNA extraction, purification columns are recommended, due to their high efficiency and standardization. Additionally, the preamplification step can be performed to increase the concentration of complementary DNA (cDNA) in the samples without impairment of sensitivity. Trizol-based method available for miRNA extraction is an alternative to consider, but its efficiency is unclear; thus, purification columns remain the preferable approach. In order to measure miRNA levels in biological samples post-extraction, Taqman-based quantitative polymerase chain reaction (qPCR) on cDNA is considered to be a golden standard. This technique is characterized by high sensitivity and specificity, but it allows to measure few miRNAs in only one run. There are other techniques available, alternative to qPCR, like Custom Exiqon locked nucleic acid, Nanostring Technologies, or small RNA sequencing, which measure the expression profile of a large number of miRNAs per run. However, due to the high cost and preanalytical bias of those methods, qPCR remains the one recommended. To lessen the risk of the influence of technical variability on the miRNA levels measurements in qPCR, a synthetic oligonucleotide should be added as an exogenous normalizing target (Garcia et al., 2020). MiRNAs might potentially replace currently used platelet function tests and provide us with more specific and reliable data on platelet activity.

## Micrornas as Regulators of Physiological Platelet Reactivity

The process of platelet activation, leading to the degranulation, changes in platelet shape, and expression of surface molecules, depends on the interaction of numerous receptors and biological pathways; therefore, it may be modified in various ways (van der Meijden and Heemskerk, 2019). MiRNAs can regulate the phenotype of platelets and modify their function by targeting the expression of specific proteins essential for platelet physiology. MiRNA expression profile differs between stimulated and resting platelets (Osman and Fälker, 2011). Previous studies showed multiple correlations between specific miRNAs and the expression of proteins participating in platelet activation and aggregation (Osman and Fälker, 2011; Kaudewitz et al., 2016; Liu et al., 2019). Moreover, activated platelets may shed miRNAs, which upon internalization may affect protein expression in other cells (Gidlöf et al., 2013; Laffont et al., 2013). In order to discuss the influence of antiplatelet drugs on platelet-related miRNA levels, it is important to take the physiological interactions between platelets and miRNAs into consideration.

### miRNAs and Receptors Related With Platelet Aggregation Pathways

#### MiR-34b-3p

Platelets may be activated *via* thromboxane receptors stimulated by TxA2 produced from AA by cyclooxygenase 1 (COX-1). COX-1 is the therapeutic target of ASA, which irreversibly binds and inactivates the enzyme. The novel research found that the expression of thromboxane A synthase 1 (TBXAS1) may be directly affected by miR-34b-3p. Inhibition of miR-34b-3p in megakaryocytes increased their viability and decreased the expression of thromboxane synthase and thromboxane B2, a stable metabolite of TxA2 (Liu et al., 2019). The platelet thromboxane pathway is mediated by the COX-1 activity, but the relation of platelet miRNAs with this enzyme needs further research.

#### MiR-19b-1-5p

Mir-19b-1-5p was associated with thromboxane-mediated platelet aggregation. MiR-19b-1-5p was suspected to be a regulator of NO-cGMP signaling pathway since *in silico* it was found to target *GUCY1A3*, *NOS3*, and *PDE5* genes (Singh et al., 2021). *GUCY1A3* encodes a subunit of soluble guanylyl cyclase, an enzyme vital for the NO-cGMP pathway (Wobst et al., 2015; Kessler et al., 2017). One of cGMP functions is the inhibition of platelet aggregation, thus the *GUCY1A3* genetic variant associated with decrease of sCG and impaired production of cGMP reduced platelet reactivity after exposure to NO (Kessler et al., 2017). However, the influence of miR-19b-1-5p on NO-cGMP-dependent platelet activation needs to be further explained.

#### MiR-223

One of the most studied miRNAs, miR-223, was shown to be abundantly present in platelets and involved in their reactivity (Landry et al., 2009; Osman and Fälker, 2011), but it is worth noting that it is also highly expressed in cells produced by hematopoietic system and endothelium (Ramkissoon et al., 2006; Haneklaus et al., 2013; Shi L. et al., 2013). Nonetheless, it directly affects 3’UTR of P2Y_12_ mRNA, regulating the expression of this ADP receptor essential for platelet aggregation (Landry et al., 2009). Moreover, thrombin-activated platelets release miR-223 in complexes with Ago2, the protein important for miRNA-mediated actions, which are internalized by endothelial cells, where they can modify genes and protein expression (Laffont et al., 2013).

#### MiR-126

Another highly expressed platelet miRNA that is suspected to impact platelet activation and aggregation on multiple levels is miR-126. Similar to miR-223, miR-126 can also play a role in the modification of the expression of P2Y_12_ receptors (Kaudewitz et al., 2016; Zhou et al., 2019), but additionally, it is suggested that it may influence platelets at early development stages. In megakaryoblastic cells, there is a correlation between miR-126 and its target gene—*ADAM9*, coding the protein potentially involved in platelet-to-collagen I adhesion and collagen-induced platelet activation (Cominetti et al., 2009; Kaudewitz et al., 2016). Although miR-126 was not proven to be involved in the differentiation of human-derived megakaryocytes, their transfection with miR-126 resulted in an increase of P-selectin expression in platelet-like structures after thrombin stimulation (Kaudewitz et al., 2016; Garcia et al., 2019) which comes together with their more procoagulant properties and increased ability of thrombin generation (Zapilko et al., 2020). Studies also revealed that miR-126-3p can directly affect the *PLXNB2* gene, resulting in downregulation of proteins encoded by this gene. PLXNB2 belongs to the family of plexins, which are receptors for semaphorins and play a role in platelets actin dynamics and thrombus formation. Thus, miR-126 can influence the platelet reactivity on many levels, mainly through the regulation of their surface proteins (Kaudewitz et al., 2016; Garcia et al., 2019).

#### MiR-376c

Since platelets may be activated *via* numerous pathways including several types of receptors, like protease-activated receptor 4 (PAR4), which is a membrane receptor for thrombin. Gene ontology analysis revealed a correlation between PAR4 activity and a gene encoding phosphatidylcholine transfer protein (PTCP), a protein participating in PAR4-mediated platelet function. It was observed that enhanced activation of PAR4 receptors stimulated platelet PTCP expression. MiR-376c targeted and inhibited PTCP mRNA and its levels were inversely correlated with PAR4 activity. Thus, PTCP expression was reduced due to the increased miR-376c expression (Edelstein et al., 2013).

#### MiR-96

Activated platelets release granules, thus hyperreactive platelets demonstrated an increase of vesicle associated membrane protein 8 (VAMP8)—a protein critical for degranulation. MiR-96 was found to bind on the 3’ UTR region of *VAMP8*. The overexpression of miR-96 correlated with a decrease of both VAMP8 mRNA and protein levels, suggesting a regulatory function of miR-96 on VAMP8 and granule secretion (Kondkar et al., 2010).

#### MiR-15a, miR-339-3 p, miR-365, miR-495, miR-98, and miR-361-3p

Several miRNAs, namely, miR-15a, miR-339-3 p, miR-365, miR-495, miR-98, and miR-361-3p, were found as the most variably expressed in platelets stimulated by thrombin. Further *in silico* analysis for target prediction found that the most important genes from the neurotrophin and mTOR signaling pathways correlated with the identified miRNAs and platelet function (Osman and Fälker, 2011). It is known that the mTOR cascade plays an important role in glycoprotein GPVI-mediated platelet aggregation (Aslan et al., 2011). MiR-15a-5p was shown to be a direct regulator of five genes (*FYN, SRGN, FCER1G, MYLK*, and *PRKCQ*) involved in the GPVI signaling pathway, participating in collagen-induced activation. Megakaryocytes transfected with miR-15a-5p exhibited lowered collagen-related protein-induced αIIbβ3 activation, and no changes were observed upon stimulation by thrombin or ADP (Basak et al., 2019). Further studies are needed to confirm other predicted miRNA target pathways in human platelets.

#### MiRNA-27b

Platelets participate not only in hemostasis but also in a number of other biological processes, e.g., inflammation, fibrosis, and angiogenesis; thus some other miRNAs not related with platelet aggregation are described as related to the platelet function in this review (see Supplementary Table 1). MiR-27b expressed in platelets targets pro-angiogenic TSP-1 (thrombospondin 1) mRNA and inhibits its translation. Thrombin activation leads to the downregulation of platelet-derived miR-27b, which can result in the significant upregulation of thrombin-induced TSP-1 expression in platelets. The overexpression of miR-27b blocked TSP-1 synthesis, leading to the increased platelet pro-angiogenic activity, which suggests that miR-27b might be a regulator of platelet-mediated angiogenesis by altering protein synthesis in activated platelets (Miao et al., 2018).

#### MiRNA-320b

**FIGURE 1:**
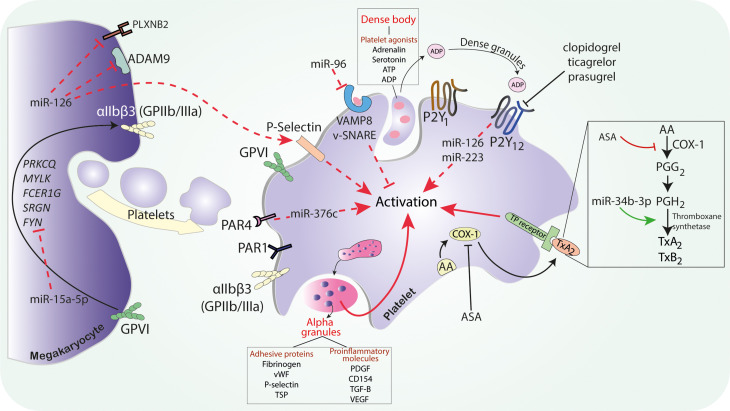
The possible mechanism of miRNAs on platelet activation. Abbreviations: ASA, acetylsalicylic acid; ADAM9, ADAM metallopeptidase domain 9; ADP, adenosine diphosphate; ATP, adenosine triphosphate; AA, arachidonic acid; COX, cyclooxygenase; FCER1G, Fc fragment of IgE receptor Ig; GPIIb/IIIa, glycoprotein IIb/IIIa; miR, microRNA; MYLK, myosin light chain kinase; PDGF, platelet-derived growth factor; PLXNB2, plexin-B2; PGG2, prostaglandin G2; PGH2, prostaglandin H2; PAR, protease activated receptor; PRKCQ, protein kinase C theta; SRGN, serglycin; SNARE, soluble NSF attachment protein receptor; SNARE, soluble NSF attachment protein receptor; TGF-B, transforming growth factor B; TSP-1, thrombospondin 1; TxA2, thromboxane A2; TP, thromboxane receptor; VEGF, vascular endothelial growth factor; VAMP, vesicle associated membrane protein; vWF, von Willebrand factor.

As it was mentioned, platelet-derived miRNAs may regulate the activity of other cells. Activated platelets affected endothelial function by shedding miR-320b, described as a regulator of the endothelial intercellular adhesion molecule 1 expression (Gidlöf et al., 2013). As it was mentioned that some types of miRNAs may be expressed by various types of cells, miR-320b was also found to be released by pancreatic cells, keratinocytes, or ovarian cells (Machtinger et al., 2017; Wang et al., 2018; Jingyang et al., 2020). This fact is important when designing studies focused on the assessment of miRNAs as specific biomarkers. Up to date different expression of a number of miRNAs and their potential correlation with platelet reactivity were presented, but their specific role in platelet physiology is yet to be identified (Figure 1 and Supplementary Table 1).

## Microrna Studies Linked With Antiplatelet Treatment

**FIGURE 2:**
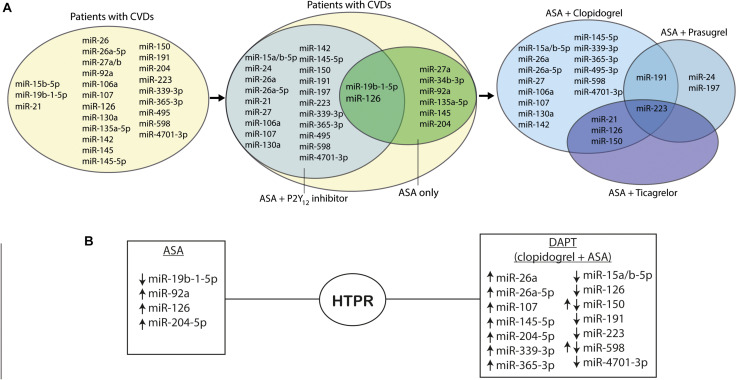
MiRNAs in platelet function. **(A)** Venn diagrams presenting regulation of miRNAs in platelet reactivity. **(B)** MiRNAs regulations in high on-treatment platelet reactivity; ↑ indicates upregulation of the miRNAs, and ↓ indicates downregulation of the miRNAs. Abbreviations: miR, microRNA; CVDs, cardiovascular diseases, ASA, acetylsalicylic acid; P2Y_12_ inhibitor, purinergic receptor inhibitor; HTPR, high on-treatment platelet reactivity.

Platelet inhibition plays a pivotal role in the prevention of atherothrombotic events, thus antiplatelet drugs are frequently administered in acute coronary syndrome (ACS), ischemic stroke (IS), and other cardiovascular diseases (CVDs) patients. Dual antiplatelet therapy (DAPT) is the standard treatment for attenuating platelet function in ACS and after percutaneous coronary intervention (PCI) in stable coronary disease (Valgimigli et al., 2018). According to the ESC guidelines, DAPT consists of ASA and one of the P2Y_12_ receptor inhibitors, preferably ticagrelor or prasugrel. Clopidogrel, as a weaker, variably acting, platelet P2Y_12_ inhibitor may be administered when ticagrelor and prasugrel are contraindicated, poorly tolerated, or if the therapy is inaccessible (Collet et al., 2020). However, adverse cardiovascular events such as myocardial reinfarction or stent thrombosis (ST) are still observed in some patients despite DAPT. Several possible causes were put forward to explain the phenomenon of ischemic events in patients on antiplatelet treatment, such as genetic polymorphisms, drug–drug interactions, or HTPR (Buonamici et al., 2007; Russo et al., 2016). HTPR observed during both ASA and P2Y_12_ receptor antagonists therapy was associated with a higher risk of adverse cardiovascular events (Buonamici et al., 2007; Aradi et al., 2010; Dannenberg et al., 2020). As was previously described, miRNAs are able to modify the expression of platelet proteins by targeting mRNAs and thereby altering their biochemical pathways including those associated with drug response. The explanation of molecular mechanisms of platelet reactivity and their impact on antiplatelet treatment efficacy seems to be important due to the large number of patients requiring antiplatelet therapy (Parker et al., 2020) (Figures 1, 2).

### MicroRNA Expression and Platelet Reactivity During ASA Treatment

Acetylsalicylic acid is the most commonly prescribed antiplatelet drug for the secondary prevention of cardiovascular events (Byrne and Colleran, 2020). Although routine monitoring of the ASA therapy is not recommended, it may benefit in some cases, e.g., to assess treatment adherence, explain the causes of therapeutic failure, or monitor the risk of bleeding incidents (Lordkipanidzé, 2012; Orme et al., 2017). A worsened efficacy of ASA prevention was also observed in overweight and obese patients, probably due to the insufficient TxA2 synthesis inhibition on standard doses (Patrono and Rocca, 2017; Rothwell et al., 2018). The latest study revealed a strong correlation between insulin resistance and the risk of HTPR during antiplatelet therapy with ASA (Jia et al., 2021). The results indicated that the evaluation of antithrombotic effects may be beneficial in a high risk population, but it is necessary to develop an accurate method with confirmed clinical usefulness for such evaluation (Rothwell et al., 2018; Jia et al., 2021). The research works performed so far have found miRNAs that may be expressed differently during ASA administration and proposed them as potential biomarkers of HTPR on ASA. It is worth noting that the majority of previous studies assessed changes in miRNAs levels during ASA therapy without investigating the molecular basis of their possible action. The evidence of a direct influence of miRNA on the thromboxane pathway is limited. It was described that miR-34b-3p is correlated with the expression of *TBXAS1*. Moreover, in patients treated with ASA, *TBXAS1* expression was higher in a group with HTPR measured in LTA, but the correlation with miR-34b-3p could not be assessed possibly due to its low abundance in plasma (Liu et al., 2019). Contrary to that, another research described differences in miRNAs expression correlated with platelet reactivity in ASA-treated patients, but it should be noted that the effect of other factors, unrelated to ASA mechanism of action, such as chronic endothelial damage, circulating lipoprotein levels, or even advanced age, may be responsible for the observed results (Nazari-Jahantigh et al., 2012; Ho et al., 2020; Lo Curto et al., 2021). MiR-126 is one of the most thoroughly studied miRNAs in relation to platelet reactivity on ASA treatment. Platelets have been demonstrated to be an important source of circulating miR-126, but it is worth emphasizing that it is profusely released also by the endothelium (Nazari-Jahantigh et al., 2012). However, it was shown that platelets activated *in vitro* by AA released miR-126, which was also inhibited by ASA. In addition, platelet reactivity measured by P-selectin expression in the group of DM patients was significantly correlated with circulating miR-126 levels. The administration of ASA for the CVDs prevention in this group led to the subsequent inhibition of platelet reactivity and the decrease of circulating miR-126. Thus, miR-126 may be considered as a potential biomarker for assessing ASA treatment efficacy (de Boer et al., 2013). Interestingly, a single nucleotide polymorphism facilitating pri-miR-126 processing correlated with the increased levels of platelet activation markers. As it was previously described, miR-126 is known to inhibit the expression of *ADAM9*, a protease that can impact the antiplatelet drug response by cleaving membrane proteins and modulating P2Y_12_ receptor synthesis (Kaudewitz et al., 2016). Although ASA affects platelet activation *via* TxA receptor by inhibiting TxA2 synthesis, P2Y_12_ receptor is also critical for this pathway (Armstrong et al., 2011). It was suggested that miR-126 can impact the platelet adhesion to collagen, while the downregulation of miR-126 release due to ASA administration correlates strongly with impaired platelet activity (Kaudewitz et al., 2016). Although in this review we tend to focus on antiplatelet treatment, it is worth noting that specific miRNAs may also be considered in further research focusing on biomarkers of antithrombotic therapy safety and efficacy. This suggestion of different application of miRNA comes from the correlation observed between overexpression of miR-126 and increased platelet-mediated thrombin generation in ASA-treated patients (Zapilko et al., 2020). According to the COMPASS trial results, CVDs patients treated with low-dose anticoagulant rivaroxaban with ASA presented better cardiovascular outcomes than those treated with ASA alone. However, combined antiplatelet and anticoagulation treatment was associated with more major bleeding events, thus the benefit/risk ratio should be considered carefully (Eikelboom et al., 2017). Described correlation between miR-126 and thrombin generation indicates that miRNAs may be helpful in tailoring the antithrombotic therapy in CVDs patients (Zapilko et al., 2020).

One of the *in vitro* analyses revealed that the expression of several miRNAs, namely, miR-1225-3p, miR-1271, miR-1537-5p, miR-19b-1-5p, miR-548e, and miR-587, was correlated with platelet aggregation reduced by indomethacin, mimicking ASA therapy in platelets collected from healthy individuals (Kok et al., 2016). However, further validation confirmed that only the downregulation of miR-19b-1-5p is potentially associated with increased platelet reactivity on ASA in isolated platelet samples. It may suggest that miR-19b-1-5p can be a potential biomarker of HTPR on ASA, and thus useful for evaluating the risk of recurrent thrombotic events (Kok et al., 2016). In line with these findings, recent results show the strong correlation between lower platelet miR-19b-1-5p and increased AA-induced platelet aggregation associated with ASA mechanism of action during DAPT (Singh et al., 2021). As it was mentioned, a potentially existing relationship between miR-19b-1-5p and modulation of NO-cGMP signaling pathway promoted by ASA may play an important role and needs to be investigated. Another strategy to assess potential pathways modulating platelet reactivity was an integrative network-based approach identification of the gene–gene interaction network and its relation with targeting miRNAs in patients with CVDs on ASA. Based on a complex multi-omics analysis network, seven genes (i.e., *TSP-1, CDC42, CORO1C, SPTBN1*, *TPM3*, *GNL2*, and *MAPRE2*) were predicted as targets for two miRNAs, namely, miR-135a-5p and miR-204-5p, which were related to platelet reactivity in ASA-treated patients (Zufferey et al., 2016). The potential role of miR-135a-5p in platelet function is yet to be studied, but the association of miR-204-5p and increased platelet reactivity was confirmed in ACS patients on DAPT therapy. In addition, the bioinformatic analysis revealed that miR-204-5p may be related to platelet microvesicles release and the formation of platelet synapsis (Ding et al., 2019). Another miRNA associated with platelet reactivity on ASA therapy, namely, miR-92a, was analyzed in the group of peripheral artery disease patients. It was found that the platelet distribution width/miR-92a ratio was correlated with platelet reactivity measured with ASPI test during ASA therapy irrespective of dose and may serve as a potential predictive tool in the clinical practice (Binderup et al., 2016). Furthermore, ASA treatment stimulated the expression of miR-27a, enhancing the P-glycoprotein (P-gp) synthesis (Paseban et al., 2020). P-gp is crucial for the thienopyridines absorption, e.g., P2Y_12_ receptor inhibitors—clopidogrel, prasugrel, and ticlopidine (Mega et al., 2010; Cattaneo, 2011). It can be hypothesized that ASA administration may decrease the absorption of P2Y_12_ receptor inhibitors *via* the upregulation of P-gp synthesis mediated by miR-27a (Paseban et al., 2020). To summarize, out of the miRNAs assessed in the conducted studies, the strongest correlation with platelet reactivity on ASA treatment was observed for miR-34b-3p, miR-19b-1-5p, miR-135a-5p, miR-204-5p, miR-126, and miR-92a suggesting their possible usefulness as biomarkers of HTPR (de Boer et al., 2013; Binderup et al., 2016; Kok et al., 2016; Zufferey et al., 2016; Ding et al., 2019; Liu et al., 2019). Nonetheless, the association between ASA mechanisms of action and changes of miRNAs expression remains unknown and needs to be investigated, taking into account that ASA may interfere with other than platelet-derived miRNAs. For example, an *in vitro* experiment showed the upregulation of miR-145 expression in vascular smooth muscle cells treated with ASA, correlated with the reduction of CD40, the protein stimulated by proinflammatory TNF-α expression (Guo et al., 2016). When planning research, it should be borne in mind that the anti-inflammatory and antiproliferative effects of ASA may also affect the level of circulating miRNAs (Figure 2).

### MiRNA Expression in Patients Receiving DAPT—Focus on ASA and Clopidogrel Combination

The formation of active clopidogrel metabolite depends on different variables like genetic, clinical, and pharmacological factors and, as one of the components, it leads to interindividual differences in the antiplatelet treatment response and HTPR. Patients with HTPR are known to present with a significantly higher risk of CV death, MI, and ST post-PCI (Aradi et al., 2010). Despite many disadvantages of clopidogrel administration, newer P2Y12 inhibitors—prasugrel and ticagrelor, often come with increased risk of bleeding, which in some patients, leads to the need to de-escalate the therapy with clopidogrel (Patti et al., 2020). Thus, monitoring of patients on DAPT with clopidogrel, more comprehensive understanding of interindividual difference in response to clopidogrel treatment, together with more advanced platelet-function monitoring techniques, could be beneficial in the prevention of adverse thrombotic outcomes (Aradi et al., 2010).

In patients receiving DAPT consisting of ASA and clopidogrel, miR-223 was the most studied miRNA, but exact correlation of this miRNA with antiplatelet treatment still remains unclear (Shi R. et al., 2013). In diabetic patients on DAPT with coronary heart disease who underwent PCI, lower platelet reactivity was associated with higher expression of miR-223, both in platelets and in plasma. In patients with HTPR on clopidogrel therapy, platelet miR-223 was significantly decreased (Shi R. et al., 2013). Similar findings were revealed in non-ST elevation myocardial infarction (NSTEMI) patients treated with clopidogrel and ASA. In this group, levels of miR-223 in plasma correlated with platelet reactivity index (PRI) and were substantially associated with the lower level of platelet inhibition on clopidogrel (Zhang et al., 2014). Decreased plasma levels of miR-223 together with the downregulation of miR-126 were also found to be related with HTPR in both healthy subjects and ACS patients receiving DAPT consisting of ASA and clopidogrel (Liu et al., 2020).

Contrary to the findings mentioned above in another study, decreased levels of miR-223 along with miR-126, miR-150, and miR-191 were found to be associated with more potent platelet inhibition on antiplatelet therapy. Among all studied miRNAs, miR-223 was the most differentially expressed in PRP when compared to serum and platelet-poor plasma (PPP) (Willeit et al., 2013). Interestingly, Chen et al. did not find miR-223 as a potential biomarker of HTPR on antiplatelet treatment, although it was correlated with the SYNTAX score, and thereby with coronary artery disease (CAD) severity.

The abovementioned findings suggest that miR-223 is an important factor in the mechanism of platelet reactivity and response to antiplatelet treatment, which indicates potential usefulness of miR-223 in the diagnosis of patients with insufficient platelet inhibition on DAPT consisting of clopidogrel and ASA. However, the results of the studies were contradictory—in some studies, platelet activation inhibition increased the miR-223 expression, whereas in other studies, the levels of miR-223 were found decreased. Additionally, some studies could not find significant changes of miR-223 due to antiplatelet therapy. These contradictory results might be related, i.e., to different origins of miR-223—as it was mentioned, not only platelets are the source of miRNAs in the blood, thus more research is required to assess the clinical usefulness and predictive value of specific platelet-related miRNAs.

Not only miR-223, but also other miRNAs were investigated as potential indicators of DAPT efficacy. MiR-339-3p and miR-365-3p were found as most expressed miRNAs in hyperreactive platelets. Additionally, miR-365-3p was characterized by the highest specificity and sensitivity in detecting HTPR on antiplatelet therapy indicating the need for its further evaluation as a tool for the monitoring of therapeutic response (Chen et al., 2019). In another cohort of both healthy subjects and ACS patients receiving DAPT consisting of ASA and clopidogrel, increased expression of miR-150 associated with HTPR was observed, which may be related to its role in the megakaryocyte-erythrocyte progenitor differentiation (Liu et al., 2020). Expression of platelet miR-26a was significantly higher in patients in whom the antiplatelet treatment with clopidogrel resulted in lower platelet inhibition when compared to patients with normal platelet inhibition and healthy cohorts. Moreover, it was found that VASP protein expression was significantly upregulated in patients with HTPR and miR-26a might target VASP mRNA, although the direct mechanism of this interaction is not clear. To conclude, platelet miR-26a was found to play a role in platelet reactivity during clopidogrel therapy. Combining information on miRNA expression and VASP PRI tests might be beneficial for platelet function evaluation in patients treated with DAPT (Chen et al., 2016).

Platelet miRs were also shown as involved in clopidogrel treatment safety. The interactions between miRNAs, mRNAs, and drug-related toxicity in response to clopidogrel treatment were analyzed *in silico* using expression profile data from patients, which were treated with ASA and clopidogrel. It was suggested that in ACS patients with HTPR, modulation in expression of specific miRNAs (upregulation of miR-145-5p and miR-26a-5p and downregulation of miR-107, miR-15b-5p, miR-4701-3p, and miR-598) may influence the expression of target genes (*ST13, BTNL3, CFD, SLC7A8*, and *SENP5*)—which could lead to clopidogrel-related liver and renal injury. Moreover, miRNAs–mRNAs interactions were associated with liver and kidney damage-related to clopidogrel response. However, clinical validation of the results described above is essential. Therefore, the differentially expressed miRNAs mentioned in this review may represent important biomarkers for clopidogrel safety and efficacy. The miRNAs described in Freitas et al.’s (2016) study may help to explain the pathways involved in the results of the treatment with clopidogrel and its possible adverse effects.

The extent of platelet inhibition by specific antiplatelet treatment combinations can modulate the miRNAs expression, but one may not exclude the impact of other drugs affecting the results (Zhang et al., 2014; Chen et al., 2019). Undoubtedly, long clinical studies are required for the assessment of the clinical usefulness of miRNAs in the HTPR detection.

### MicroRNA Expression in Patients Receiving Different DAPT Regimens

According to the recent European guidelines, DAPT consisting of ASA and ticagrelor or prasugrel is preferred to the combination with clopidogrel (Collet et al., 2020) largely due to high interindividual variability of platelet inhibition during the administration of recommended clopidogrel doses, while ticagrelor and prasugrel strongly inhibit platelet reactivity in standard dosing regimens (Aradi et al., 2014). The monitoring of HTPR is recommended mostly for clopidogrel-treated patients to stratify the risk of ST, but it is not a well-established practice in the group receiving ticagrelor or prasugrel, though it may be equally important to identify the patients treated with newer P2Y_12_ antagonists from the perspective of high risk of bleeding (Aradi et al., 2014, 2015). It is worth noting that in most cases, the comparison was made between different DAPT regimens to assess the influence of P2Y_12_ inhibitors. In patients treated with DAPT consisting of ASA and ticagrelor or prasugrel, miR-223 increase in plasma correlated significantly with decreased ADP-induced platelet reactivity. The results indicate that miR-223 expression may reflect the level of platelet inhibition during P2Y_12_ inhibitor therapy (Chyrchel et al., 2015). High expression of miR-223 was found to serve as a potential independent predictive marker of perioperative bleeding in off-pump coronary artery bypass surgery. The levels of this miRNA were significantly higher in patients treated with DAPT consisting of ASA and ticagrelor when compared to those treated with ASA and clopidogrel (Wang et al., 2020). Not only miR-223, but also miR-150, miR-21, and miR-126 expressions during DAPT were strongly correlated with the type of administered P2Y_12_ receptor inhibitor. Their circulating levels were increased in ticagrelor-treated patients compared with clopidogrel and prasugrel groups. Interestingly, drug cessation did not influence miRNAs expression despite a significant increase in ADP receptor−mediated platelet activation (Jäger et al., 2019). However, it should be noted that rebound phenomenon and increased platelet reactivity after the antiplatelet drug withdrawal was previously described (Lordkipanidzé et al., 2009). Thus, the studied miRNAs seem difficult to apply as a biomarker of platelet reactivity in this scenario. On the contrary, replacing clopidogrel by ticagrelor in DAPT resulted in the significant inhibition of platelet aggregation and the downregulation of miR-126, miR-223, and miR-150 levels as well as the upregulation of miR-96 in plasma. In line with the previous study, drug cessation did not significantly affect the miRNAs expression (Carino et al., 2016). Despite the rebound phenomenon, it may be speculated that platelets are not the only source of circulating miRNAs, e.g., miR-126 is also released by the endothelium (Lordkipanidzé et al., 2009). Another study focusing on miR-150 and miR-21 associated with platelets and extracellular vesicles in groups treated with three different DAPT regimens revealed that PEVs release was not correlated with platelet reactivity and did not depend on the P2Y_12_ inhibitor type. However, treatment cessation resulted in significant changes in relations between PEVs and miR-150 and miR-21 expressions. This result suggests that during all regimes of DAPT, platelets may release the same amount of PEVs, but their miRNA content is altered (Haller et al., 2020). Only one study compared the effects of clopidogrel, prasugrel, and ASA monotherapy showing that diabetes mellitus (DM) patients receiving prasugrel had lower expression of miR-223, miR-24, miR-191, and miR-197 compared to the group treated with ASA only. No significant differences were observed in miRNA levels among patients treated with ASA vs clopidogrel or clopidogrel vs prasugrel. Moreover, miR-197 levels were decreased in patients receiving ASA or prasugrel with CVDs when compared to patients with no CVDs. Clopidogrel-treated groups did not demonstrate similar results. In addition, the downregulation of miR-197 was suggested to be correlated with an increased risk of AMI. All in all, there is an association between miR-21, miR-24, miR-197, miR-223, and miR-126 levels, and platelet reactivity. Those miRNAs, together with miR-197, are known to be reduced by antiplatelet therapy in healthy volunteers, as well as in patients with CVDs. The results of the study indicate that, in the case of DM patients, prasugrel monotherapy may deliver better results in platelet inhibition measured by ADP aggregation, P-selectin expression, and collagen-induced activation than ASA or clopidogrel administration (Parker et al., 2020) (Figure 2).

Further studies with a larger cohort are necessary to better understand the role of circulating miRNAs in platelet reactivity on DAPT and how their expression is correlated with clinical outcomes to finally assess their usefulness as a biomarker.

## Platelet-Derived miRNA and Genetic Variability in Antiplatelet Treatment

Genetic and epigenetic variability may influence the efficacy and safety of antiplatelet treatment (Miao et al., 2018). Approximately 30% of patients treated with conventional dose of clopidogrel present HTPR during therapy (Cirillo et al., 2020). Clopidogrel is a prodrug metabolized by cytochrome P450—CYP1A2, CYP2C19, and CYP2B6 into 2-oxo-clopidogrel. Further, CYP3A4, CYP3A5, CYP2C9, CYP2C19, and CYP2B6 convert it to an active form, which irreversibly binds to the platelet P2Y_12_ receptor (Sangkuhl et al., 2010). It was reported that 12% of response variability is due to CYP2C19 polymorphism and as much as 88% of the causes of different platelet inhibition levels remain unclear (Shuldiner et al., 2009). Hence, it is important to emphasize that genetic and epigenetic variability may affect the efficacy and safety of antiplatelet treatment (Miao et al., 2018).

As mentioned before, CYP2C19 plays an important role in clopidogrel metabolism. Its polymorphisms, which lead to functional differences in the enzyme action, could be an important factor to be taken into consideration for the planning of antiplatelet therapy containing clopidogrel (Jiang et al., 2015). The most common enzymes encoding alleles, which present with decreased activity, are CYP2C19^∗^2, in which there is a transition of guanine (G) into adenine (A) at position 681 (rs4244285) and CYP2C19^∗^3, in which the transition from G to A occurs at position 636 (Dehbozorgi et al., 2018). This knowledge helps us to understand the complex correlations between enzymes polymorphisms, miRNAs expression, and response to treatment with clopidogrel.

The presence of CYP2C19^∗^2 isoform can reduce the conversion of clopidogrel from prodrug to active metabolite, which corresponds with diminished antiplatelet responsiveness to clopidogrel—where patients with CYP2C19^∗^2 rs4244285 AA/AG allelic variants are considered as poor metabolizers, while GG variants are associated with normal metabolism (Fontana et al., 2007; Zhang et al., 2015; Yi et al., 2016). Among patients with ACS treated with clopidogrel for 1 year, CYP2C19^∗^2 rs4244285 AA genotype was significantly associated with increased risk of acute myocardial infarction (AMI) and CYP2C19^∗^3 rs4986893 AG genotype with the risk of ST. Moreover, patients that were carriers of two CYP2C19 loss of function (LOF) alleles had a significantly increased risk of ST and AMI when compared to only one LOF carrier or non-carrier. This phenomenon may be caused by the reduced enzymes activity in CYP2C19^∗^2 and CYP2C19^∗^3 LOF polymorphisms carriers, impaired conversion of clopidogrel into an active metabolite, and, what follows, insufficient P2Y_12_ inhibition (Zhou et al., 2020).

It is suggested that miRNAs may play a role in the mechanism of poor response to clopidogrel therapy among patients with specific CYP alleles. As it was mentioned before, patients with CYP2C19^∗^2 rs4244285 AA allelic variants are poor metabolizers (Yi et al., 2016) and this might be related to the action of miR-1343-3p and miR-6783-3p, which bind to the A allele of the CYP2C19 rs4244285 gene and possibly lead to the downregulation of CYP2C19 rs4244285 AA protein expression (Sharma et al., 2020).

Moreover, the correlation between miR-223, miR-221, and miR-21 expression levels and the clopidogrel response in patients with CYP2C19^∗^2 genotype was found. As it was previously described, those miRNAs are predicted to impact the antiplatelet treatment *via* targeting the 3’-UTR of the human P2Y_12_ receptor mRNA. Levels of platelet miR-223, miR-221, and miR-21 were found to be significantly higher in extreme high responders compared to low responders. However, this result may be impacted by the interaction with CYP2C19^∗^2 genotype. Further analyses showed that the increased levels of platelet-derived miRNAs (miR-223, miR-221, and miR-21) were positively correlated with enhanced efficacy of clopidogrel antiplatelet responsiveness in ACS patients. This relationship could be found only in CYP2C19^∗^2 carriers, but not in CYP2C19^∗^2 non-carriers, probably due to worsened clopidogrel efficacy caused by impaired metabolism to an active form. In non-carriers, this effect may be masked by a higher concentration of active metabolites (Peng et al., 2017).

Lack of platelet inhibition during clopidogrel therapy can be explained not only by CYP2C19 polymorphism, but also by miRNA polymorphism. MiR-605-5p targets and inhibits the expression of mRNA of P2Y_12_ receptor, ABCB1 and CYP2B6. MiR-605-3p on the other hand targets and inhibits the CYP2B6 mRNA. Pre-miR-605 rs2043556, which is a precursor of miR-605-5p and miR-605-3p, occurs in two allelic variants—pre-miR-605-A and pre-miR-605-G. Transfection of cell lines with pre-miR-605-A plasmid results in much higher levels of miR-605 and, as a consequence, in significant inhibition of CYP2B6 and P2R_12_ mRNA transcription. Contrary to that—pre-miR-605-G plasmid and blank control deliver the same results. ACS patients with miR-605-G allelic variant present less AMI and unstable angina (UA) when compared to miR-605-A allelic patients, after 1 year of clopidogrel treatment, which may be due to lesser inhibition of CYP2B6. It is hypothesized that normal expression of P2Y_12_ receptors in miR-605-G allelic variant individuals is related to a better response to antiplatelet treatment because the appropriate amount of active drug can bind to the normal number of ADP receptors (Zhou et al., 2020).

Those findings could help to predict the antiplatelet treatment responsiveness. Personalized therapy based on genetic variations may lead to the reduction of MACE and improve treatment efficacy (Yang et al., 2015). Further studies are needed to investigate the impact of the abovementioned mechanisms in patients treated with, e.g., ticagrelor—an agent acting without active metabolite conversion by liver CYP450.

## Platelet-Derived miRNAs Associated With Higher Platelet Reactivity in Patients With Cardiovascular Diseases

In order to understand the correlations between antiplatelet drugs and miRNAs expression and apply this knowledge to optimize antiplatelet treatment, it is important to know how miRNAs can change in certain diseases, for the treatment of which antiplatelet drugs are administered. Knowledge about miRNA levels in pathological conditions could help clinicians to note changes indicating the risk of HTPR, MACE, or other therapy complications.

### Risk Factors of CAD and ACS

It should be noted that various factors can influence the synthesis of platelet-related miRNAs in patients with CVDs and they should be taken into consideration in the evaluation of miRNAs fluctuations during antiplatelet therapy and their potential role as a diagnostic or prognostic tool. Undoubtedly, the risk factors of CVDs like DM, hyperlipidemia, smoking, hypertension, or obesity and their relation with miRNAs expression should be considered (Tzoulaki et al., 2016; Ding et al., 2017). Several miRNAs—miR-223, miR-126, and miR-140, which are important for platelet activation and aggregation processes, were found to be downregulated in platelets of patients with DM. This potentially leads to the upregulation of P2Y_12_ receptors and less potent platelet inhibition by antiplatelet drugs acting through this receptor (Fejes et al., 2017). Hypertension along with hyperlipidemia was also found to influence the expression of various platelet-related miRNAs, out of which miR-126, miR-146a, miR-223, and miR-214 were found to be significantly upregulated, while miR-143, miR-10a, and miR-145 were downregulated in platelets. Simultaneously, miR-222, miR-221, miR-34a, and miR-210 levels were increased, while miR-223 levels together with miR-146a, miR-214, miR-143, and miR-145 were decreased in PEVs (Alexandru et al., 2019). These findings are based mainly on animal studies, and further clinical research is needed to confirm their results. It is also interesting that platelet-derived miR-223 and miR-22 were significantly downregulated in hypertensive patients when compared to the normotensive control group. Additionally, levels of miR-223, miR-22, and miR-126 were significantly decreased in hypertensive patients with CVDs, thus the platelet-related miRNAs may indicate the occurrence of cardiovascular complications in these patients (Marketou et al., 2019). Smoking is another cardiovascular risk factor that impacts platelet-related miRNAs. Smokers, in comparison with the control group, presented with the decreased total number of circulating microvesicles, which may have been related with the reduced number of PEVs. The only significantly downregulated miRNA in smokers was niRNA-223 (Badrnya et al., 2014).

Increased thrombogenicity constitutes an important factor in the pathogenesis of DM and IS. Expression of both platelet-derived miR-223 and miR-146a in the cohort with DM as well as those with both DM and IS was found to be significantly lower than in healthy controls. Inverse correlation was found between high platelet activation rate and platelet miR-223 and miR-146a expression. Moreover, lower expression of these two miRNAs was associated with higher blood glucose levels. Thus, it shows a link between hyperglycemia and regulation of platelet reactivity, suggesting that hyperglycemia may downregulate miRNA expression leading to enhanced platelet reactivity. Low platelet and plasma miR-223 and miR-146a expression can thus be considered as a risk factor of the IS development in diabetic patients (Duan et al., 2014).

### ACS and MACE

In the process of ACS development, the role of platelets is essential—after the plaque rupture, they adhere to the vessel walls and become activated. Platelet activation causes the release of secondary agonists—TxA_2_ and ADP. ADP-P2Y_12_ interacts with GPIIb/IIIa receptor, enhancing its response for agonists, which leads to the formation of stable, platelet-rich thrombus. At the same time, platelets surface is coated with thrombin, which converts fibrinogen to fibrin resulting in the formation of a more stable platelet-fibrin-rich clot (Gurbel et al., 2016). It was suggested that miRNAs may serve as potential biomarkers of ACS because, among others, they can participate in the regulation of endothelial cells function, angiogenesis, plaque stabilization, cardiomyocyte differentiation, and cardiac hypertrophy. Furthermore, there is a possibility that platelet-related miRNAs influence pathological thrombosis, which contributes to the ACS development (Navickas et al., 2016; Stojkovic et al., 2019). Despite the antiplatelet therapy, patients receiving DAPT are still at risk of both thrombotic and hemorrhagic complications, which suggests that pathways leading to ACS progression may, to some extent, differ from those in primary hemostasis (Stakos et al., 2012). Currently available diagnostic methods and biomarkers are insufficient, and there is a need to seek for new, clear biomarkers that could be useful in the assessment of risk–benefit balance in antiplatelet therapy (Koupenova et al., 2017). The fact, that upon activation platelets can shed miRNAs and, what follows, affect gene expression, may suggest miRNAs as potentially useful indicators of the ongoing pathophysiological processes in the cardiovascular system (Indolfi et al., 2019; Sabatino et al., 2019; Zareba et al., 2020).

The correlation between the levels of specific miRNA previously described to be associated with platelet function, and ACS was found in the plasma of ST elevation myocardial infarction (STEMI) patients. Namely, miR-21 and miR-126 were downregulated, whereas miR-150 and miR-223 were upregulated in a cohort of patients with STEMI. The varying expressions were observed both at the time of pre-PCI and at 48 h post-PCI. Among the studied miRNAs, miR-126 exhibited the highest correlation with the plasma levels of cTnI (Li et al., 2017). The decreased platelet levels of miR-126 in ACS patients may be related to the completed thrombus formation resulting in STEMI (Kaudewitz et al., 2016; Li et al., 2017; Garcia et al., 2019). However, the study performed by Li et al. (2017) did not find an association between miRNAs and platelet reactivity (by using VerifyNow P2Y_12_ assay and VASP assay). On the contrary, another study showed that positive correlation between miRNAs and platelet reactivity markers was seen both in the general population and in ACS patients with the residual platelet reactivity during antiplatelet therapy. The study found a significant association between miR-223, miR-126, and VASP assay as well as miR-126 and VerifyNow P2Y12 assay. The inhibition of miR-126 in platelets affected expression of P2Y12 receptor and reduced platelet aggregation, which was also shown in animal models. The findings suggest that miR-126 and miR-223 can modify platelet reactivity in ACS. Moreover, the studied miRNAs are suggested to be potentially useful as ACS prognostic biomarkers (Kaudewitz et al., 2016). Also miR-22, miR-185, miR-320b, and miR-423-5p were found to be downregulated in platelets and thrombus collected from STEMI patients. Furthermore, these miRNAs were upregulated in activated platelet supernatant, which suggests the role of platelet reactivity for their release, although the phenomenon was not reflected in peripheral circulation (Gidlöf et al., 2013). In the case of NSTEMI patients, correlation between miR-15b-5p, miR-93, and miR-126 expression levels and platelet reactivity was observed (Becker et al., 2020).

Studies performed up to date suggest that specific miRNAs may be considered as potential indicators of ongoing thrombus formation (Gidlöf et al., 2013; Kaudewitz et al., 2016; Li et al., 2017). Thus, according to the study performed by Tang et al. (2019), high plasma levels of miR-142 were correlated with a significantly elevated risk of MACE during DAPT. This miRNA is suspected to be associated with platelet aggregation on clopidogrel treatment, so its levels might be considered as an indicator of HTPR. Furthermore, lower miR-19b-1-5p was shown to be associated with higher AA-mediated reactivity and higher risk of MACE in patients on DAPT after invasively treated AMI (Tang et al., 2019; Singh et al., 2021). It is worth noting that many factors may increase the risk of MACE, but the mechanisms of correlations between miRNAs, platelet reactivity, and MACE are not clear. The risk of cardiovascular events can be heightened due to a number of factors, i.e., HTPR (Buonamici et al., 2007; Aradi et al., 2010; Dannenberg et al., 2020). However, miRNAs might also influence the transcription of specific genes and lead to the abnormalities of the process of hemostasis, thrombosis, and antiplatelet drug response (Teruel-Montoya et al., 2015; Parker et al., 2020). Results of current studies are insufficient, and more research leading to comprehensive understanding of miRNA-platelets correlations could lead to clinical applications of miRNAs prognostic value. There is a possibility that cautious investigation of the changes in miRNAs expression could be beneficial not only for the explanation of pathophysiology of diseases, but also for the monitoring of antiplatelet treatment, and it might facilitate to minimize the adverse effects of therapies while enhancing the benefits.

## Conclusion

In conclusion, miRNAs are abundantly represented in platelets and may regulate platelet reactivity by targeting specific genes and modifying protein expression. Moreover, through regulation of gene expression, they can affect the biological functions of other human cells in, e.g., endothelium. The presented studies demonstrated that the selected types of platelet-derived miRNAs may be considered as diagnostic and prognostic biomarkers of HTPR; however, their clinical application should be further investigated. MiRNA-based assessment of platelet reactivity may improve the prediction of antiplatelet treatment efficacy as well as create an individual antiplatelet treatment tailored to highly specific patient needs. Previous studies revealed that individualizing of the antiplatelet therapy according to the platelet function measured by MEA provided benefits for patients after PCI (Hazarbasanov et al., 2012; Siller-Matula et al., 2013). It is worth noting that adjusting the antiplatelet therapy regimen to the VerifyNow results failed to provide any benefits (Price et al., 2011; Trenk et al., 2012; Cayla et al., 2016). Therefore, further research evaluating miRNAs as biomarkers for therapeutic drug monitoring should be based on well-established current knowledge on platelet function assessment. The presented review found the limitations impeding the analysis of the performed studies: (i) miRNAs were measured in various types of biological samples such as PPP, PRP, or isolated platelets and using different laboratory protocols, thus the results are uncomparable; (ii) some types of miRNAs expressed by platelets may also be released by different cells; (iii) the studies were conducted in populations living in the different geographical regions, which may imply genetic variabilities; (iv) platelet reactivity was measured by various uncomparable methods; and (v) only one study assessed the influence of P2Y_12_ receptor inhibitors monotherapy, therefore the precise effect of these drugs on miRNA expression is poorly understood. As we have shown, platelet-derived miRNAs can be considered as biomarkers of platelet reactivity and antiplatelet therapy efficacy, but well-designed studies are essential to establish many practical aspects such as measurement standardization, type of biological material most suitable for measurements, kinetics of miRNAs in circulation, and genetic variabilities in different populations.

## Author Contributions

PC, DJ, CE, AF, and MP contributed to the data collection and elaboration, writing, and approval of manuscript, and are guarantors of the article. AF, AG, and KF contributed to writing, editing, discussion, and approval of the manuscript. JS-M and MP contributed to supervising, revising, and approval of the manuscript. CE and ZW contributed valuable graphical designs. The corresponding author attests that all listed authors meet authorship criteria and that no others meeting the criteria have been omitted. All authors contributed to the article and approved the submitted version.

## Conflict of Interest

The authors declare that the research was conducted in the absence of any commercial or financial relationships that could be construed as a potential conflict of interest.

## Abbreviations

A: adenine
AA: arachidonic acid
ACC: American College of Cardiology
ACS: acute coronary syndrome
ADAM9: ADAM metallopeptidase domain 9
ADP: adenosine diphosphate
Ago2: argonaute 2
AHA: American Heart Association
AMI: acute myocardial infarction
ARU: aspirin reaction units
ASA: acetylsalicylic acid
ATP: adenosine triphosphate
Bid: twice a day
BTNL3: butyrophilin like 3
CAD: coronary artery disease
CCS: Canadian Cardiovascular Society
cDNA: complementary DNA
CFD: complement factor D
clo: clopidogrel
CORO1C: coronin 1C
COX: cyclooxygenase
COX-1: cyclooxygenase 1
CRP: C reactive protein
CVDs: cardiovascular diseases
CYP: cytochrome P450
d: day
DAPT: dual antiplatelet therapy
DM: diabetes mellitus
DNA: deoxyribonucleic acid
ECG: electrocardiogram
eNOS: endothelial NOS
ESC: The European Society of Cardiology
FCER1G: Fc fragment OfIgE receptor Ig
G: guanine
GPIIb/IIIa: glycoprotein IIb/IIIa
GPVI: platelet glycoprotein 4
GUCY1A3: guanylate cyclase soluble subunit alpha-3
HC: healthy control
HTPR: high on-treatment platelet reactivity
HUVEC: human umbilical vein endothelial cells
IS: ischemic stroke
ISTH: International Society on Thrombosis and Hemostasis
LDP: leukocyte-depleted platelets
LOF: loss of function
LPR: low platelet reactivity
LTA: light transmittance aggregometry
MACE: major adverse cardiovascular events
MACCE: major adverse cardio − cerebrovascular
MAPRE2: microtubule associated protein RP/EB family member 2
MEA: multiple electrode aggregometry
miR, MicroRNA: miRNA
mRNA: messenger RNA
mTOR: mammalian target of rapamycin
MYLK: myosin light chain kinase
NGS: next generation sequencing
NO: nitric oxide
NO-cGMP: cyclic guanosine monophosphate
NOS: nitric oxide synthase
NSTE-ACS: non-ST elevation acute coronary syndrome
NSTEMI: non-ST elevation myocardial infarction
PAG: ADP-induced platelet aggregation
PAR1-AP: proteinase-activated receptor 1 activation peptide
PAR4: protease-activated receptor 4
PCI: percutaneous coronary intervention
PCTP: phosphatidylcholine transfer protein
PEVs: platelet-derived extracellular vesicles
PFP: platelet-free plasma
P-gp: P-glycoprotein
PGG2: prostaglandin G2
PLXNB2: Plexin-B2
PPP: platelet-poor plasma
PRI: platelet reactivity index
PRKCQ: protein kinase C theta
PRP: platelet-rich plasma
PRU: platelet reactivity units
RI: relative platelet inhibition
RT-qPCR: reverse transcription quantitative polymerase chain reaction
SENP5: SUMO specific peptidase 5
SLC7A8: solute carrier family 7 member 8
SPTBN1: spectrin beta, non-erythrocytic 1
SRGN: serglycin
ST: stent thrombosis
STEMI: ST elevation myocardial infarction
ST13: suppression of tumorigenicity 13
TBXAS1: thromboxane A synthase 1
Tica: ticagrelor
TNF-α: tumor necrosis factor α
TP: thromboxane receptor
TPM3: tropomyosin 3
TSP-1: thrombospondin 1
TxA2: thromboxane A2
UA: unstable angina
3’UTR: 3’ untranslated region
VAMP: vesicle associated membrane protein
VASP-P: vasodilator-stimulated phosphoprotein-phosphorylation
VEGF: vascular endothelial growth factor
vWF: von Willebrand factor
wks: weeks.
