# Supplementary material for: MicroRNA as Potential Biomarkers of Platelet Function on Antiplatelet Therapy: A Review
Source: Front Physiol. 2021 Apr 15;12:652579. doi: 10.3389/fphys.2021.652579 (PMC8081881; doi:10.3389/fphys.2021.652579)
Supplement: Supplementary file 1 [file Table_1.docx]

**Table 1.** Studies evaluating miRNAs as diagnostic/prognostic biomarkers and therapeutic potential in platelet reactivity

| Ref | miRNAs expression | Material/ study type | Methodology platelet reactivity and miRNA analysis | Number of patients/controls and inclusion criteria | Therapeutic scheme | Conclusion |
| --- | --- | --- | --- | --- | --- | --- |
| [(Chen et al., 2016)](https://paperpile.com/c/lnu8kJ/1uDiB) | ↑miR-26a | LDP/ Human, *in silico* | PRI was measured by VASP phosphorylation in a flow cytometry (PLTVASP/P2Y12 kit; Diagnostica Stago S.A.S., Paris, France);  RT-qPCR. | 43/20    - 45 - 80 years old;  - CAD diagnosis;  -planned stent implantation; | clo 300 mg loading dose + 75 mg/d (no data about ASA but patients were after PCI) | Platelet expression of miRNA-26a was associated with high platelet reactivity during DAPT. |
| [(Shi et al., 2013b)](https://paperpile.com/c/lnu8kJ/re2Xg) | ↓miR-223 | LDP/ Human | PRI was measured by VASP phosphorylation (PLT VASP/P2Y12 kit, BIOCYTEX, France), in a flow cytometry (Beckman Coulter, Miami, FL, USA),  ADP-induced PAG measured by light transmission aggregometry;  RT-qPCR. | 33 (17 normal/16 low responders);    - CAD patients with NSTE-ACS (prolonged (>20 min) angina pain at rest; new onset (de novo) angina (CCS II/III);  - non-diabetic;  - recent destabilization of previously stable angina (CCS III);  - ischemic changes  on the ECG; | Received a loading dose  of 300 mg aspirin plus 300 mg clopidogrel for at least 24 h, or 100 mg  ASA plus 75 mg clopidogrel for at least 5 days. Platelet reactivity after clopidogrel loading dose (300 mg) was determined | Decreased miR-223 expression was the independent predictor associated with the presence of PRI-determined HTPR. |
| [(Kondkar et al., 2010)](https://paperpile.com/c/lnu8kJ/Ptnfy) | ↓miR-96 | LDP/ Human, *in silico* | Epinephrine aggregation;  RT-qPCR | 288    - healthy subjects;  - European Americans and African Americans; | - | Overexpression of miR-96 caused a dose-dependent decrease in VAMP8 protein and mRNA, suggesting a role in VAMP8 mRNA degradation. |
| [(Osman and Fälker, 2011)](https://paperpile.com/c/lnu8kJ/afdj7) | ↓miR-15a, ↓miR-339-3, ↑miR-365, ↑miR-495, ↑miR-98, ↑miR-361-3p | LDP/ Human, *in silico* | n/d;  RT-qPCR | 6    - healthy blood donors; | - | Thrombin stimulation was associated with altered levels of some miRNAs in platelets. |
| [(Gidlöf et al., 2013)](https://paperpile.com/c/lnu8kJ/SP27z) | ↑miR-22,  ↑miR-185,  ↑miR-320b, ↑miR-423-5p  (in platelets supernatant)  ↓miR-22,  ↓miR-185,  ↓miR-320b, ↓miR-423-5p  in platelets | LDP/ human | n/d;  RT-qPCR | 10/15    - STEMI (diagnoses were based on ECG criteria); | - | Released platelet miR-320b was shown to have a paracrine role on endothelial cell intercellular adhesion molecule-1 expression. |
| [(Duan et al., 2014)](https://paperpile.com/c/lnu8kJ/2O4pB) | ↓miR-223, ↓miR-146a | LDP, PPP/ human | Flow cytometric analysis of the surface expression of P-selectin;  RT-qPCR | 7 (DM), 6 (IS), 6 (DM+IS)/8 (HC)    - without a history of using antiplatelet or antidiabetic drugs;  - DM diagnosed first time;  - patients with IS and DM - diabetes diagnosed right after acute IS;  - stroke patients with large artery atherosclerosis; |  | Hyperglycemia may downregulate the expressions of miR-223 and miR-146a, leading to subsequent platelet activation. |
| [(Chyrchel et al., 2015)](https://paperpile.com/c/lnu8kJ/hDoaB) | ↓miR-223 | Plasma/human | MEA (Multiplate analyzer, Dynabyte, Münich, Germany) to assess platelets aggregation in response to ADP;  RT-qPCR | 21 (10 ticagrelor or prasugrel / 11 clopidogrel)    - male;  - CAD admitted to NSTEMI with an uncomplicated hospital course;  - From the day of admission, the patients were receiving either clopidogrel (n=11) or prasugrel/ticagrelor (n=10) in addition to ASA | 1) ASA (75-100 mg/d) + clo (300-600 mg load + 75 mg/d); 2) ASA (75-100 mg/d) + prasugrel (60 mg load + 10 mg/d); 3) ASA (75-100 mg/d) + tica (180 mg load + 90 mg/bid) | More potent platelet inhibition associated mainly with newer P2Y12 antagonists appears to coincide with higher miR-223 relative to the subjects with attenuated responsiveness to DAPT. |
| [(Willeit et al., 2013)](https://paperpile.com/c/lnu8kJ/cbd7Y) | ↑miR-126,  ↑miR-223,  ↑miR-150,  ↑miR-191 | platelets, microvesicles, PRP, PPP, serum/ Human, *in silico* | Platelet aggregometry, formation of thromboxane A2 (measured as thromboxane B2), VerifyNow;  RT-qPCR | - healthy men <40 years old (n=19);  - DM patients (n=19);  - carotid atherosclerosis patients (n=33); | dose-escalation study in healthy volunteers at 4 different time points: at baseline without therapy, at 1 wk with 10 mg prasugrel, at 2 wks with 10 mg prasugrel + 75 mg ASA, and at 3 wks with 10 mg prasugrel + 300 mg ASA. Pharmacological intervention in patients: 75 mg of ASA at baseline. Eight were randomized and received dipyridamole and 4 clopidogrel in addition to ASA. | Platelets have substantial contribution to the circulating miRNA pool and identified miRNAs (miR-24, miR-197, miR-191, and miR-223) decreased during  antiplatelet therapy. |
| [(Binderup et al., 2016)](https://paperpile.com/c/lnu8kJ/GhhFl) | ↑miR-92a | PPP/ human | MEA (Multiplate® Analyzer, Roche Diagnostics, Rotkreuz, Switzerland), platelets aggregation was stimulated by ARA, collagen, TRAP-6-peptide, ristocetin, and ADP, ASA resistance was defined according to ASPI test;  RT-qPCR | 50/50    - 50 healthy blood donors and 50 patients with intermittent claudication;  - treated with ASA; | ASA (75 mg, 100 mg or 150 mg/d) as monotherapy, which they confirmed to have been taking for at least 10 days before blood sampling | HTPR on ASA can potentially be identified by miR-92a levels in plasma combined with PDW. |
| [(Peng et al., 2017)](https://paperpile.com/c/lnu8kJ/ODhuE) | ↓miR-223,  ↓miR-221,  ↓miR-21 | LDP/ Human, *in silico* | RI measured by LTA (APACT-4 aggregometer LABiTec, Ahrensburg, Germany):  RT-qPCR | 21 (extremely high-responders) / 18 (extremely low-responders)    - ACS diagnosis (including UA, STEMI, NSTEMI) according to the AHA/ACC criteria; | Before clopidogrel administration and after 5 days’ maintenance dose of clopidogrel  administration, 20 μmol/L ADP induced platelet aggregation was measured for each patient | Platelet-derived miRNAs (miR-223, miR-221 and miR-21) are independently associated with platelet reactivity on  clopidogrel therapy. However, the association could be influenced by the interaction with CYP2C19*2 genotype |
| [(Freitas et al., 2016)](https://paperpile.com/c/lnu8kJ/iwjyh) | ↑miR-145-5, ↑miR-26a-5p  ↓miR-107, ↓miR-15b-5, ↓miR-4701-3p, ↓miR-598 | peripheral blood/Human, *in silico* | Platelet reactivity was evaluated by VerifyNow® ASA and P2Y12 assays; | 8 (4 high reactive platelets/  4 low reactive platelets) | ASA (100 md/d) + clo (75 mg/d) | Differential expression of miRNAs in platelets and interactions with their target mRNAs are associated with variability in platelet reactivity, clopidogrel response and drug-induced toxicity. |
| [(Kok et al., 2016)](https://paperpile.com/c/lnu8kJ/1m36b) | ↓miR-19b-1-5p | isolated platelets/ Human | Platelet aggregation initiated by ADP (Multiplate® Analyzer, Roche) measured in same samples with or without indomethacin; RT-qPCR | 10/15    - 35 - 65 years old;  - ASA and simvastatin therapy;  - no personal or family history of cardiovascular disease; | 100 mg of ASA/d, for 2 wks. Since this cohort was also intended as a control group for subjects with CAD, all subjects received simvastatin 40 mg/d, for 6 wks, of which the last 2 wks in addition to ASA | Lower platelet miR-19b-1-5p expression is related to high platelet reactivity on ASA. |
| [(Carino et al., 2016)](https://paperpile.com/c/lnu8kJ/5ndw5) | ↑miR-126, ↑miR-223, ↑miR-150,  ↓miR-96 | Plasma/ human | Whole blood platelet aggregometry (Multiplate® Analyzer, Roche);  RT-qPCR | 8/25 (with load)  8/25 (without load)    - diagnosed with ACS;  - treated with DAPT (ASA+clopidogrel or ASA+ticagrelor) | ASA 100 mg/d + clo 75 mg/d; after switch ASA 100 mg/d + tica 90 mg/bid; patients from 'load' group received additionally 180 mg loading tica dose | Switching from a dual antiplatelet treatment with clopidogrel to ticagrelor is associated with significant modulation in the circulating levels of specific microRNAs. |
| [(Tang et al., 2019)](https://paperpile.com/c/lnu8kJ/1YnM9) | ↑miR-126-5p,  ↑miR-130a-3p,  ↑miR-142-5p,  ↑miR-27a-3p,  ↑miR-106a-5p,  ↑miR-21-5p | Plasma/ human | ARU, PRU verification by VerifyNow, RT-qPCR | 115 + 1230    - Han Chinese;  - 40 and 80 years old;  - diagnosed with CAD;  - treated with DAPT (ASA+clopidogrel); | DAPT of clopidogrel and ASA after PCI surgery, 115 CAD patients | Six miRNAs (miR-126, miR-130a, miR-27a, miR-106a, miR-21, and miR-142)  were associated with clopidogrel-treated platelet aggregation, but only a high plasma miR-142 level were independent risk factors of MACE. |
| [(Zhang et al., 2014)](https://paperpile.com/c/lnu8kJ/LCLOy) | ↓miR-223 | Plasma/ human | PRI measured by VASP (PLT VASP/ P2Y12 kit, BIOCYTEX, France) phosphorylation in flow cytometry (Cytomics FC500, Beckman Coulter, Miami, FL, USA);  RT-qPCR | 31 normal-responders/31 low-responders    - Chinese Han;  - diagnosed with NSTE-ACS patients with troponin negative (normal routine troponin testing at least 12 h from symptom onset) presenting;  - treated with DAPT (ASA+clopidogrel); | loading dose of 300 mg ASA + 300 mg clopidogrel for at least 24 h, or 100 mg ASA plus 75 mg clopidogrel for at least 5 days | Decreased miR-223 was associated with higher platelet reactivity on clopidogrel. |
| [(Edelstein et al., 2013)](https://paperpile.com/c/lnu8kJ/PdSmE) | ↓miR-376c | LDP, PRP/ Human, *in silico* | LTA was used to assess maximal percent aggregation in PRP treated with ARA, ADP, anti-CD9, CRP, PAR1-AP, \PAR4-AP. LTA to assess thrombin induced platelet aggregation. Assessment of calcium mobilization, profiling and qRT-PCR validation | 70/84    - healthy subjects;  - black and white;  - non-diabetic; | - | *MiR-376c* levels were differentially expressed by race and PAR4 reactivity, and were inversely correlated with *PCTP* mRNA levels, PC-TP protein levels and PAR4 reactivity |
| [(de Boer et al., 2013)](https://paperpile.com/c/lnu8kJ/fcJsF) | ↑miR-126,  ↑miR-16,  ↑miR-223,  ↑miR-423 | PRP, PFP/ human, | Measurement of P-selectin after incubation with ARA (AA, 1.5 mmol/L, Hart Biologicals, UK) in the absence or presence of ASA; RT-qPCR | 40/4    - DM patients;  - ASA treatment (100 or 300 mg/day) or placebo; | - | ASA inhibited *in vitro* platelet activation and the transfer of miR-126 from the platelet to the plasma compartment. *In vivo* administration of ASA resulted both in platelet inhibition and reduction of circulating levels of platelet-derived microRNAs including miR-126. |
| [(Laffont et al., 2013)](https://paperpile.com/c/lnu8kJ/w7ksx) | ↑miR-223 | Platelet-derived MPs, HUVEC/ *in vivo* (HUVEC) | Flow cytometry (Canto II SORP BD Biosciences) was used to determine platelet activation status in freshly isolated and thrombin activated platelets; RT-qPCR | - healthy volunteers; | - | Platelets activated with thrombin preferentially release their miR-223 content in MPs. These MPs can be internalized by endothelial cells and regulate their gene expression. |
| [(Kaudewitz et al., 2016)](https://paperpile.com/c/lnu8kJ/Ng8Z4) | ↑miR-126,  ↑miR-223,  ↑miR-24,  ↑miR-191 | PPP,  LDP/ human, mice C57BL | Platelet aggregation response to ADP was determined using LTA (BioData PAP-8E optical aggregometer). PRU was recorded with VerifyNow (Accumetrics, USA). PRI was determined using VASP phosphorylation assay kits (BioCytex, France);  NGS, qRT-PCR | 125/669    - Patients with a history of ACS 30 days previously (STEMI, NSTEMI or unstable angina);  - detailed assessment of platelet function; | clopidogrel or prasugrel as maintenance therapy for the 1st group or prescribed ticagrelor as maintenance therapy for the 2nd group | Levels of platelet-related plasma miRNAs correlate with platelet function tests in patients with ACS and platelet activation markers in the general population. |
| [(Miao et al., 2018)](https://paperpile.com/c/lnu8kJ/yvVW0) | ↓miR-27b | purified platelets/ human | Platelet activation was monitored by P-selectin  expression, profiling and RT-qPCR | 32    - healthy volunteers;  - aged between 22 and 52 years | - | Thrombin stimulation *in vitro* altered platelet microRNA profiles, including decreased miR‐27b. |
| [(Zufferey et al., 2016)](https://paperpile.com/c/lnu8kJ/qrCSA) | ↑hsa-miR-1, ↑hsa-miR-656,↑hsa-miR-133,  ↑hsa-miR-410,  ↓hsa-miR-150, ↓hsa-miR-135,  ↓hsa-miR-144,  ↓hsa-miR-451,  ↓hsa-miR-128,  ↓hsa-miR-204,  ↓hsa-miR-634,  ↓hsa-miR-424 | PRP/ Human, *in silico* | LTA (aggregation on epinephrine, ARA, ADP, and collagen); profiling | 12  - symptomatic atherothrombotic disease (CAD), ischemic cerebrovascular disease and/or peripheral artery disease);  - treated with ASA 100 mg/day; | 100 mg/day of non enteric-coated ASA | miR-135a-5p and miR-204-5p was correlated with platelet reactivty in ASA-treated patients |
| [(Garcia et al., 2019)](https://paperpile.com/c/lnu8kJ/We57X) | ↑miR-126-3p | PLS/ *in vitro* (human CD34+ cells) | n/d, qPCR, protein quantification and a reporter  gene assay | - | - | Megakaryocytes transfected with miR-126-3p generated platelet-like structures exhibiting 156% more reactivity than control. PLXNB2 was validated as a new gene target of miR-126-3p in |
| [(Chen et al., 2019)](https://paperpile.com/c/lnu8kJ/I912M) | ↑miR-365-3p,  ↑miR-495-3p,  ↓miR-107, ↓miR-223-3p,  ↓miR-15a-5p,  ↓miR-96-5p, ↓miR-339-3p | PRP/ Human, *in silico* | PRU was determined using VerifyNow (Accumetrics, San Diego, CA, USA); RT-qPCR | 155/20    - patients with stable angina;  - received an elective stent implant;  - treated with DAPT consisting of ASA plus either clopidogrel, clopidogrel and cilostazol, or ticagrelor; | 1) ASA (300 mg load + 100 mg/d) + clo (300 mg load + 75 mg/d); 2) ASA (300 mg load + 100 mg/d) + tica (180 mg load + 90 mg/bid); 3) ASA (300 mg load + 100 mg/d) + clo (300 mg load + 75 mg/d) + cilostazol 100 mg/bid | MiR-339-3p and miR-365-3p had the highest sensitivity and specificity for detecting HTPR |
| [(Liu et al., 2020)](https://paperpile.com/c/lnu8kJ/ADfT) | ↓miR-223, ↓miR-126, ↑miR-150 | LDP | Platelet reactivity was measured by a thromboelastogram (TEG) through calculation of the TEG-MA_ADP_ | 214 qualified healthy volunteers (age: 38.1 ± 12.3 years, male: 45%) + 430 patients with ACS were recruited | All patients with ACS received 100 mg/d of ASA + 75 mg/d of clo after admission.  For the patients undergoing PCI, a loading dose of 300 mg of clo  followed by 75 mg/d was administered. | Significantly decreased expression of the platelet-derived miR-223 and miR-126 and increased miR-150 was detected in extremely HTPR subjects. Low levels of platelet-derived miR-223 and miR-126 could be predictive for a suboptimal antiplatelet response of clopidogrel. |
| [(Singh et al., 2021)](https://paperpile.com/c/lnu8kJ/R2zL) | ↓miR-19b-1-5p | whole blood, buffy coat | Platelet reactivity was measured using whole blood impedance aggregometry on a Multiple analyzer (Roche Diagnostics, Basel, Switzerland) | Patients (n=945) on DAPT; 72 hours after AMI treated with invasive management | All patients received DAPT (no detailed data) | Lower miR‐19b‐1‐5p expression was found to be associated with sustained platelet aggregation on ASA, and a higher risk of MACCE in patients with ACS. |
| [(Liu et al., 2019)](https://paperpile.com/c/lnu8kJ/PJuX) | ↓miR-34b-3p | whole blood/ *in vitro* Dami cells | LTA of AA-induced platelet aggregation (LBYNJ4, PRECIL, China) | Patients (n=113) with CAD, 50 years old and more, | ASA 100 mg/d | MiR-34b-3p may regulate the platelet response to ASA by suppressing TBXAS1 expression and megakaryocyte proliferation. |
| [(Zapilko et al., 2020)](https://paperpile.com/c/lnu8kJ/tBkv) | ↑miR-126 | *in vitro* platelet-like structures derived from human MKs;  *in vivo* plasma | Thrombin-antithrombin complexes (TAT) and prothrombin fragment F1 + 2 (F1 + 2), were quantified in citrated plasma samples using Enzygnost® TAT micro and Enzygnostat F1 + 2 kits (Siemens Healthcare Diagnostics Products, Marburg, Germany) | Patients (n=185) with CVDs | ASA (no detailed data) | Plasma levels of miR-126, correlated with markers of *in vivo* thrombin generation in cardiovascular patients. The results support a key role for miR-126 in platelet-supported thrombin generation. |
| [(Parker et al., 2020)](https://paperpile.com/c/lnu8kJ/L0YR) | ↓miR-24, ↓miR-191, ↓miR-197 ↓miR-223 | PRP, PPP/ human | LTA with ADP, AA and collagen and a PAP-8 aggregometer (v2.0, Bio/Data Corporation, Horsham, PA, USA), flow cytometry, qRT-PCR | 56 patients with DM | antiplatelet monotherapy with ASA 75 mg/d, clo 75 mg/d or prasugrel 10 mg/d | Potent P2Y12 inhibition  with prasugrel reduced detectable levels of miR-24,  miR-191, miR-197 and miR-223 compared to ASA. |
